# Long-term outcomes in childhood-onset systemic lupus erythematosus: preliminary results of the CHILL-NL study

**DOI:** 10.1186/1546-0096-12-S1-P311

**Published:** 2014-09-17

**Authors:** Noortje Groot, Sylvia Kamphuis

**Affiliations:** 1 Paediatrics, University Medical Centre Utrecht, Utrecht, Netherlands; 2 Pediatric Immunology/Infectious diseases/Rheumatology, Erasmus MC-Sophia, Rotterdam, Netherlands

## Introduction

Childhood-onset systemic lupus erythematosus (cSLE) is a more severe disease when compared to adult-onset SLE, with higher disease activity at onset and during disease course, higher percentage of major organ involvement and more rapid accrual of damage. Long-term outcome studies on cSLE are rare.

## Objectives

This study aims to describe the long-term outcomes of patients with cSLE that have reached adulthood.

## Methods

All Dutch rheumatologists, immunologists, nephrologists, haematologists, neurologists were asked to refer cSLE patients to the CHILL-NL (CHILdhood Lupus in the NetherLands) study team. The study was promoted in magazines and on websites of the Dutch SLE patient organizations. Interested adult cSLE patients were asked to approach the study team. All patients were seen for a single study visit. Patients’ current health status was assessed with an extensive medical interview and physical examination. All previous medical correspondence was retrieved, including information regarding cumulative multisystem involvement, auto-antibody profiles, drug use and comorbidities. SLE disease activity index-2K (SLEDAI-2K) and SLICC damage index (SDI) was calculated. Quality of life and related factors such as educational and work status, fecundity, fatigue and depressive symptoms were assessed.

## Results

31 patients (87% female) are currently included with a median age at diagnosis of 13 years (range 9-18) and median disease duration at time of visit was 15 years (range 4 – 36). At time of visit, median SLEDAI-score was 4 (0 – 9). 20/31 (65%) patients had an SDI-score of at least 1 (median 1, range 0 – 7). Current medication use included hydroxychloroquine in 63% of all patients, prednisone in 52%. 48% of the patients were treated with at least one DMARD (MMF (27%), Azathioprine (25%) and others). Half of the patients ever had CNS involvement. Two thirds of patients ever had renal involvement. Of these patients, 33% had current proteinuria (**>50 mg/mmol**). Four (13%) patients had a renal transplantation. During their disease course, 32% of the patients had been hospitalised at least once due to a severe infection. Analyses on factors related to quality of life are pending. Table 1.

**Table 1:** 

Characteristic	Frequency (median + range)	
**Age at diagnosis**	13 years (9 – 18)	

**Disease duration**	15 years (4 – 36)	

**SLEDAI-score**	4 (0 – 9)	

**SDI**	1 (0 – 7)	

**Cumulative organ involvement**		
Skin	90%	
Muskuloskeletal	83%	
Haematological	83%	
Renal	65%	
Central nervous system	48%	
Pulmonary	46%	
Cardiovascular	36%	
Abdominal	19%	
Peripheral nervous system	10%	

**(Vertebral) fractures**	23%	

**Severe infections (hospitalization necessary)**	32%	

**Medication use**	**Current**	**Ever**
Prednisone	52%	100%
Azathioprine	25%	64%
Cyclophosphamide	0%	36%
Hydroxychloroquine	63%	85%
Rituximab	0%	11%
MMF	27%	46%

**Family history**	**1^st^degree**	**2^nd^degree**
SLE	14%	0%
other auto-immune diseases	55%	57%

## Conclusion

cSLE is associated with significant long-term consequences, including frequent renal and CNS involvement, accrual of damage in the majority of patients and almost all patients still using DMARDs, prednisone, hydroxychloroquine or combinations thereof.

## Disclosure of interest

None declared

